# Particulate Organic Matter Affects Soil Nitrogen Mineralization under Two Crop Rotation Systems

**DOI:** 10.1371/journal.pone.0143835

**Published:** 2015-12-08

**Authors:** Rongyan Bu, Jianwei Lu, Tao Ren, Bo Liu, Xiaokun Li, Rihuan Cong

**Affiliations:** 1 College of Resources and Environment, Huazhong Agricultural University, Wuhan, China; 2 Key Laboratory of Arable Land Conservation, Middle and Lower Reaches of Yangtze River, Ministry of Agriculture, Wuhan, China; University of Vigo, SPAIN

## Abstract

Changes in the quantity and/or quality of soil labile organic matter between and after different types of cultivation system could play a dominant role in soil nitrogen (N) mineralization. The quantity and quality of particulate organic matter (POM) and potentially mineralizable-N (PMN) contents were measured in soils from 16 paired rice-rapeseed (RR)/cotton-rapeseed (CR) rotations sites in Hubei province, central China. Then four paired soils encompassing low (10th percentile), intermediate (25th and 75th percentiles), and high (90th percentile) levels of soil PMN were selected to further study the effects of POM on soil N mineralization by quantifying the net N mineralization in original soils and soils from which POM was removed. Both soil POM carbon (POM-C) and N (POM-N) contents were 45.8% and 55.8% higher under the RR rotation compared to the CR rotation, respectively. The PMN contents were highly correlated with the POM contents. The PMN and microbial biomass N (MBN) contents concurrently and significantly decreased when POM was removed. The reduction rate of PMN was positively correlated with changes in MBN after the removal of POM. The reduction rates of PMN and MBN after POM removal are lower under RR rotations (38.0% and 16.3%, respectively) than CR rotations (45.6% and 19.5%, respectively). Furthermore, infrared spectroscopy indicated that compounds with low-bioavailability accumulated (*e*.*g*., aromatic recalcitrant materials) in the soil POM fraction under the RR rotation but not under the CR rotation. The results of the present study demonstrated that POM plays a vital role in soil N mineralization under different rotation systems. The discrepancy between POM content and composition resulting from different crop rotation systems caused differences in N mineralization in soils.

## Introduction

Given favorable growth conditions, soil organic matter (SOM) mineralization can account for 50–80% of N accumulation by crops [1]. Thus, understanding the dynamics and mechanisms of soil N mineralization is a prerequisite for optimizing N fertilizer management, achieving higher crop yields and decreasing environmental risk [2, 3]. Mineralization of SOM is a complex biochemical process that is affected by many factors, including environmental conditions [4], indigenous soil properties [5], soil microbial communities [6] and agricultural practices [7]. In particular, the chemical composition and nature of SOM are regarded as key factors in the regulation of soil N mineralization [8]. Soil organic matter, especially labile SOM fraction, plays a dominant role in N mineralization because it serves as an easily accessible energy source for microorganisms and results in greater soil N mineralization [9]. Previous studies have indicated that soil SOM fractions, such as the light fraction [10] and labile humic acid fraction [11], serve as sinks for soil N. Removing the light fraction significantly improved the soil microbial biomass N (MBN), gross N mineralization and gross nitrification rate [12]. By contrast, Ros *et al*. [13] reported that labile SOM fractions acted as the source of soil mineralizable N. Decreases in the soil N mineralization potential occurred when dissolved organic matter was removed [14]. Indeed, the role of labile SOM in N mineralization is remains obscure.

Particulate organic matter (POM), a type of SOM fraction according to particle size analysis, has been demonstrated to be a useful tool in process-oriented SOM research because SOM in the sand-sized fraction (>53 μm) is generally more labile than SOM in the clay- and silt-sized fractions (< 53 μm) [15]. Fractions such as the POM typically represent most decomposed plant residues in the early stages of the humification process and composed, at least partially, of easily mineralizable C and N [16]. POM is important to SOM turnover and responds much faster to soil management changes than the total SOM and passive fractions [17]. Generally, soils under permanent vegetation with a large quantity of litter return have a higher proportion of POM [18]. Gosling *et al*. [19] indicated that the recent history of organic matter addition and inclusion of fallows are the primary factors controlling the POM content of agricultural soils. Some studies indicated that the pool size and chemical composition of POM is the key factor in the regulation of soil N mineralization[20]. Baldock and Skjemstad [20] found that the bulk of POM had a high content of polysaccharides. This fraction of POM, which serves as a readily decomposable substrate for soil microorganisms, is a dynamic pool of available N leading to greater soil N mineralization. However, a fraction of the POM was found that consist of biologically stable C (*e*.*g*., charcoal), which is less susceptible to decomposition [21]. The differences in POM composition associated with the sources of plant residues and their decomposition influenced the properties of the POM and soil N mineralization [22]. Olk *et al*. [23] observed that the anaerobic decomposition of incorporated rice straw residues promoted the accumulation of phenolic compounds in the soil labile organic matter and reduced the soil N supply. As more aromatic compounds accumulated in soil labile organic matter, less soil N was available for mineralization. This finding might provide a rational explanation for the aforementioned contradictory reports on the role of POM in soil N mineralization.

Crop rotation is an important agricultural practice that affects soil organic matter fractions due to the entirely distinct incorporation and decomposition conditions [24]. Compared to continuous upland rotations, repeated flooding and drying practices in paddy-upland rotations significantly affect the physical, chemical and biological properties of the soil [25]. Paddy-upland rotations have shown a larger potential for C sequestration due to perennial flooded conditions [26]. Conversion of cropland from continuous upland to paddy-upland can decrease the soil N supply, which influences crop yields [27, 28]. The reduced soil N supplied in the paddy-upland rotation was not reflected by changes in the total soil organic C and N contents, as these contents were generally higher in paddy-upland rotation systems [29, 30]. Reductions in the soil N supply following crop rotation alterations might be attributed to more changes in the soil labile organic matter fractions. Thus, we propose a hypothesis that differences in the quality and/or quantity of soil POM, caused by different rotation systems, will alter soil N mineralization. To test this hypothesis, we quantified the net N mineralization and microbial biomass size of original soils and soils from which POM was removed in typical rotation systems (i.e., rice-rapeseed and cotton-rapeseed) and evaluated the contributions of POM to N mineralization and microbial biomass size in these different rotation systems.

## Materials and Methods

### Ethics statement

No specific permits were required for these field studies. No specific permission was required for the use of these locations or performance of these activities because they were not carried out in privately owned or protected areas. The field studies did not involve endangered or protected species.

### Site description and soil sampling

Soil samples were collected from the Jianghan Plain plantation area in Hubei Province, central China (29°25′N-33°20′N, 108°21′E-116°07′E). Rice-rapeseed (RR) rotation and cotton-rapeseed (CR) rotation are the dominant cropping systems in this area, representing typical paddy-upland and upland rotation systems. The climate in this region is subtropical, with an average annual temperature of 16°C, 1,100 mm of precipitation, and a mean frost-free period of 270 days. Rice is generally transplanted in early June and harvested at the end of September. The cotton growth period is longer than the rice growth period because cotton is usually transplanted at the end of May and harvested at the end of October. Winter rapeseed seedlings (30–40 days old) were transplanted approximately one week after cotton harvest or one month after rice harvest. Winter rapeseed grows from October or November to May of the following year.

Before soil sampling, we had conducted an extensive survey on soil utilization, including soil parent material, rotation duration, tillage, fertilizer management, water and crop residues management. Based on these results, we collected 16 sites according to the methods in Mendham *et al* [31] to investigate the POM and PMN contents. In the same site, the paired samples were chosen from adjacent plots separated by a distance of less than 1 km, which were continuously planted with RR and CR rotations for at least 5 years to minimize the effects of other factors (*i*.*e*., meteorological and topographical) outside the rotation system. In addition, in order to minimize potential confounding of tillage history, according to the method of Kuzyakov and Bol [32], we select all field similar in management history. Surface soil (0–20 cm) samples aggregated from 15 soil cores were collected in early April during rapeseed season. After sampling, the soil samples were sieved, POM-separated and incubated to examine the effects of the rotation system on soil organic fractions and net N mineralization. In the following study, taking reference and borrowing from the study by Li *et al* [14], we divided 16 paired samples into four levels based on PMN content distribution, i.e., high (75–95%), low (5–25%) and two moderate levels (25–50% and 50–75%), and select four sites representing four levels to examine the effects of the POM on soil net N mineralization. Meanwhile, we sampled in triplicate to increase sample size from four selected sites which covered a wide range of soil N supply levels under two different rotation systems. The basic physical and chemical properties of the tested soils are provided in S1 Table and the average values are presented in Table 1.

**Table 1:** General properties of the soils tested.

Rotation^1^		pH^2^ (H_2_O, ratio 1:5)	SOC (g kg^-1^)	TN (g kg^-1^)	Particle size (%)^3^		
					Clay	Silt	Sand
RR	Average	6.13±0.92	15.2±3.14	1.60±0.34	27.3±9.3	59.6±8.4	13.2±13.5
	Range	4.91–7.43	9.4–20.0	1.17–2.32	13.1–43.4	49.8–74.4	0.0–34.2
CR	Average	6.00±1.05	12.1±2.89	1.34±0.23	24.4±9.0	59.6±8.8	16.0±15.2
	Range	4.38–7.53	7.4–18.1	1.03–1.72	12.0–43.2	45.1–70.0	0.6–42.9

^1^ RR: rice-rapeseed rotation; CR: cotton-rapeseed rotation.

^2^ 2.5:1 H_2_O.

^3^ Particle size categories: clay (< 0.002 mm), silt (0.02–0.002 mm), sand (0.02–2 mm).

### POM Removal Soil fractionation and analyses

POM removal was performed following the method described by Mendham *et al*. [31]. In brief, after passing the samples through a 2-mm sieve, the soil was dispersed by shaking with deionized water (3:1 water: soil ratio by weight) and then sonicated for 30 min in an ultrasonic bath at 4°C (with a sonication energy of 50 J ml^-1^). Sonication can disperse soil-labile macro-aggregates (> 250 μm), without significantly influencing other soil aggregates (< 250 μm) [33]. The subsequent soil suspension was filtered through a 53-μm sieve and rinsed again with distilled water. All the materials retained on the sieve were defined as “POM”, and the other non-particulate matter was defined as “POM-removal” [34]. The POM was oven-dried at 50°C in a glass beaker and weighed to determine the POM-C and POM-N concentrations. In addition, the non-particulate matter was air-dried for further analyses.

Total C and N were analyzed in original soil and POM fractions. Total C was determined with the K_2_Cr_2_O_7_-H_2_SO_4_ wet oxidation method, and total N content was determined with a continuous-flow auto-analyzer (AA3, Seal Analytical Inc., Southampton, UK) after digestion based on the Kjeldahl method [35].

### Infrared spectroscopy of the soil POM

The dried soil POM fraction was scanned from 4000 to 400 cm^-1^ in the MidIR range with a resolution of 4 cm^-1^ and 64 co-added scans per spectra using a Fourier transform spectrometer equipped with standard DRIFT optics under purged conditions and with a custom-fabricated sample transport system (Vertex 70, Burck Optics Inc., Ettlingen, DE) that could scan a 50 x 2 mm sample [36].

### N mineralization

An improved method following Li [14] and Mendham [29] was used to estimate the content of soil potentially mineralizable-N (PMN). Briefly, 50 g samples of the original and POM-removed air-dried soils were placed in 250-mL polyethylene bottles and their soil moisture contents were adjusted to 40% of the water-holding capacity (WHC) with deionized water. Next, the samples were aerobically incubated at 25°C for 7 days to restore the soil balance. After pre-incubation, the soil moisture was adjusted to 60% of the WHC with deionized water and aerobically incubated at 25°C for 28 days. Meanwhile, the equal amount of quartz sand (analytical reagent) of POM was re-added and mixed with the POM-removed soils to serve as a positive control for the POM-removal soil incubations. Each treatment was performed in triplicate. The soil inorganic N (NO_3_^-^-N + NH_4_^+^-N) and soil microbial biomass C (MB-C) and N (MB-N) contents were determined at 28 days after incubation. The increase in the soil inorganic N during 28 days incubation was defined as the PMN.

Inorganic N was extracted from soil by shaking fresh soil (10 g of oven-dried soil) samples with 2 mol L^-1^ KCl (1:5 soil: solution ratio) for 30 min. The extracts were then filtered (Whatman 42) to determine the NH_4_^+^-N and NO_3_^-^-N concentrations with a continuous-flow auto-analyzer (AA3, Seal Analytical Inc., Southampton, UK). The MBC and MBN contents were determined via the chloroform fumigation-extraction method [37]. The fumigated and un-fumigated soil (20 g of oven-dried soil) was shaken with 80 mL of 0.5 mol L^-1^ K_2_SO_4_ (1:4 soil: solution ratio) for 30 min and filtered. The MBC and MBN in the fumigated and un-fumigated extracts were analyzed using a total organic C analyzer (Multi N/C 3000, Agilent Technologies Inc., Jena, DE). A *k*_EN_ factor of 0.38 and a *k*_EC_ factor of 0.30 were used to calculate the MB-N and MB-C, respectively.

### Statistical analysis

The data were analyzed using the SPSS 17.0 software (SPSS Inc., Chicago, IL, USA). Datasets were tested for normality and homogeneity of variance using the Kolmogorov-Smirnov *D* and Cochran’s tests, respectively, and transformed if necessary. Significant differences in the soil POM fraction between the RR and CR rotations were compared using the paired-sample T-test with a probability level of 0.05. A multi-way analysis of variance (ANOVA) was performed to analyze the effects of POM removal, crop rotations and/or site on the soil PMN contents and soil microbial biomass C and N contents according to the factorial randomized design. Tukey’s post hoc was then applied. Linear regression analysis was used to study the relationships between the PMN and MBN contents.

## Results

### Soil POM fraction

The soil POM contents in the soils under the two different rotation systems are shown in S1 Table and the average values are presented in Table 2. No significant differences in the ratio of POM throughout the soils were observed between these two rotations (*P* = 0.143). Overall, POM accounted for 34.1% and 35.1% of the soil under the RR and CR rotations, respectively. The average POM-C and POM-N contents in the soil under the RR rotations were higher than those in the soil under the CR rotation. No significant differences were observed in the soil POM C/N ratios between these two different rotation systems (*P* = 0.481).

**Table 2:** Soil particulate organic matters contents in the different rotation systems.

Rotation	Percentage (%)	POM-C (g kg^-1^)	POM-C/SOC (%)	POM-N (g kg^-1^)	POM-N/TN (%)	POM-C/N
RR	34.2±10.5	5.68±1.5	37.9±8.1	0.51±0.16	32.2±7.6	11.7±3.9
CR	35.1±11.0	3.89±1.0	32.6±6.7	0.33±0.12	24.6±7.2	12.4±2.9
Paired T test						
*P*	0.143	<0.001	0.01	<0.001	0.001	0.481

### Potentially mineralizable-N (PMN)

**Fig 1:**
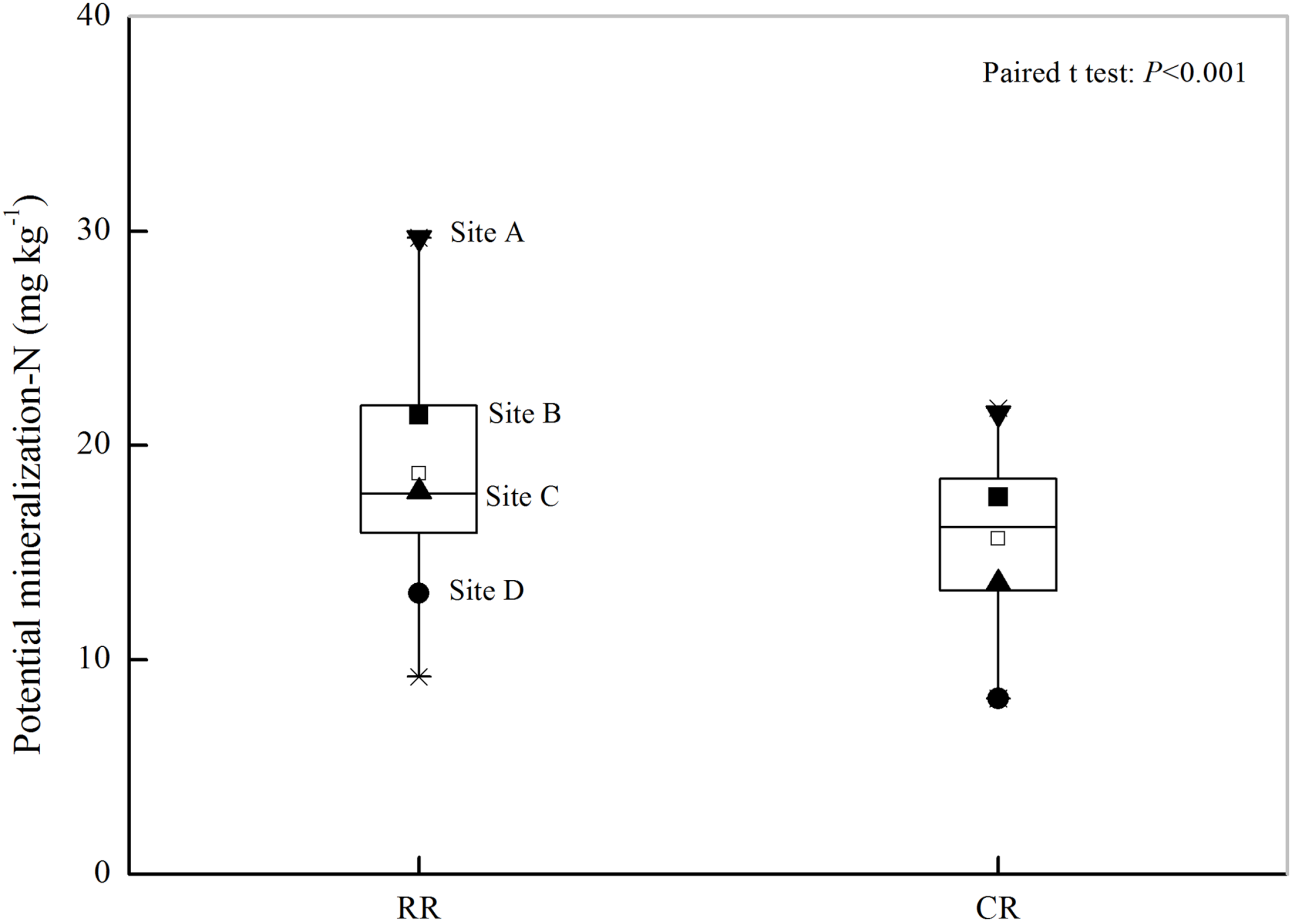
Soil potentially mineralizable N (PMN) contents under the different rotation systems. Note: RR corresponds to the rice-rapeseed rotation and CR corresponds to the cotton-rapeseed rotation. Sites A, B, C, and D represent the 4 paired soils from which particulate organic matter (POM) was removed to study the effects of POM removal on soil N mineralization.

**Fig 2:**
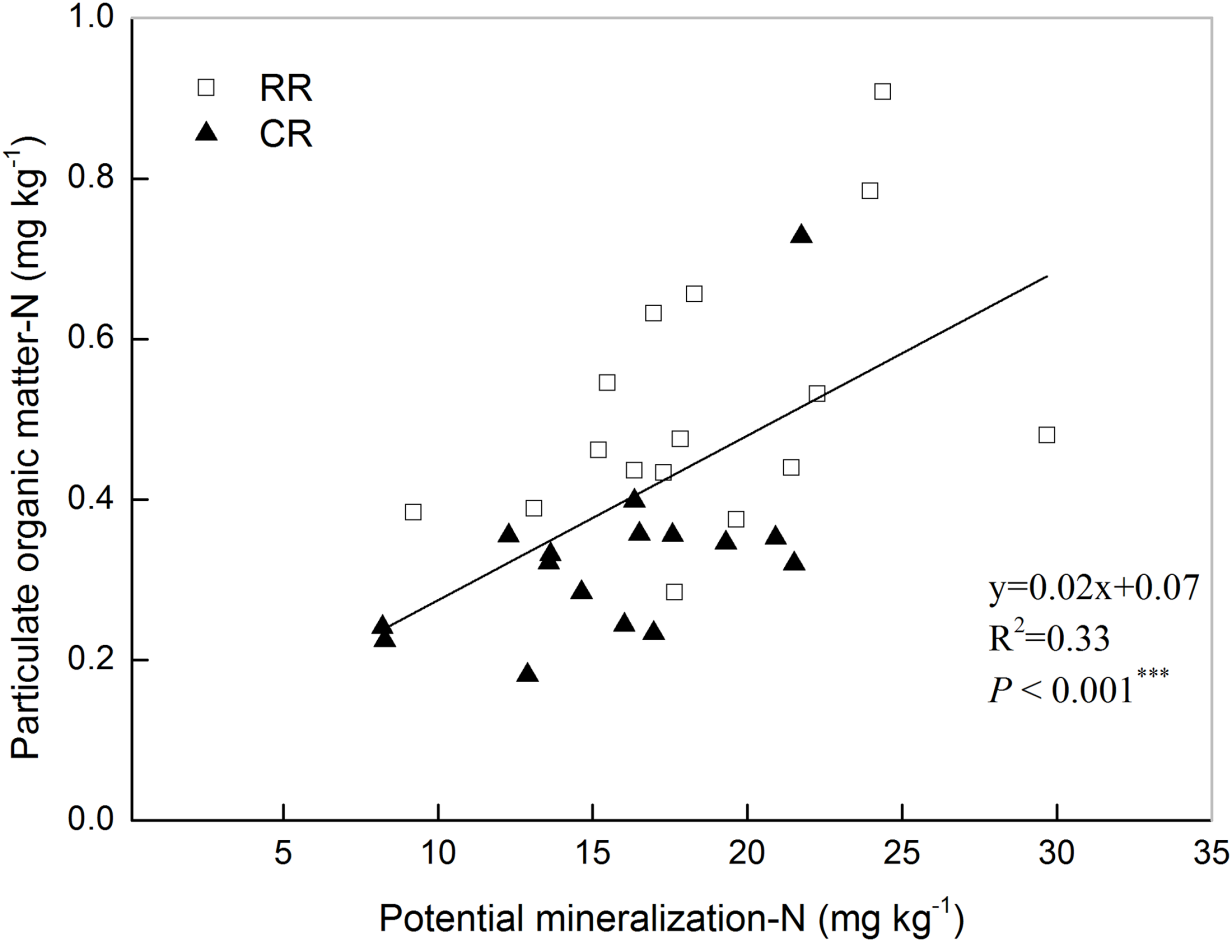
The relationships between the content of POM-N and PMN. Note: RR corresponds to the rice-rapeseed rotation, and CR corresponds to the cotton-rapeseed rotation. Bars indicate the standard error of the mean (n = 31).

Soil PMN content was significantly influenced by the rotation systems (Fig 1). Under the RR rotation, the soil PMN contents ranged from 9.21 mg kg^-1^ to 29.68 mg kg^-1^ with an average of 18.67 mg kg^-1^. The soil PMN content under the CR rotation was ranged from 8.20 mg kg^-1^ to 21.74 mg kg^-1^. The soil PMN content under the RR rotation was significantly higher than that under the CR rotation (*P* < 0.001). There was a strong positive correlation between POM and PMN contents (Fig 2, *P* < 0.001).

### Effects of POM removal on soil PMN content

**Fig 3:**
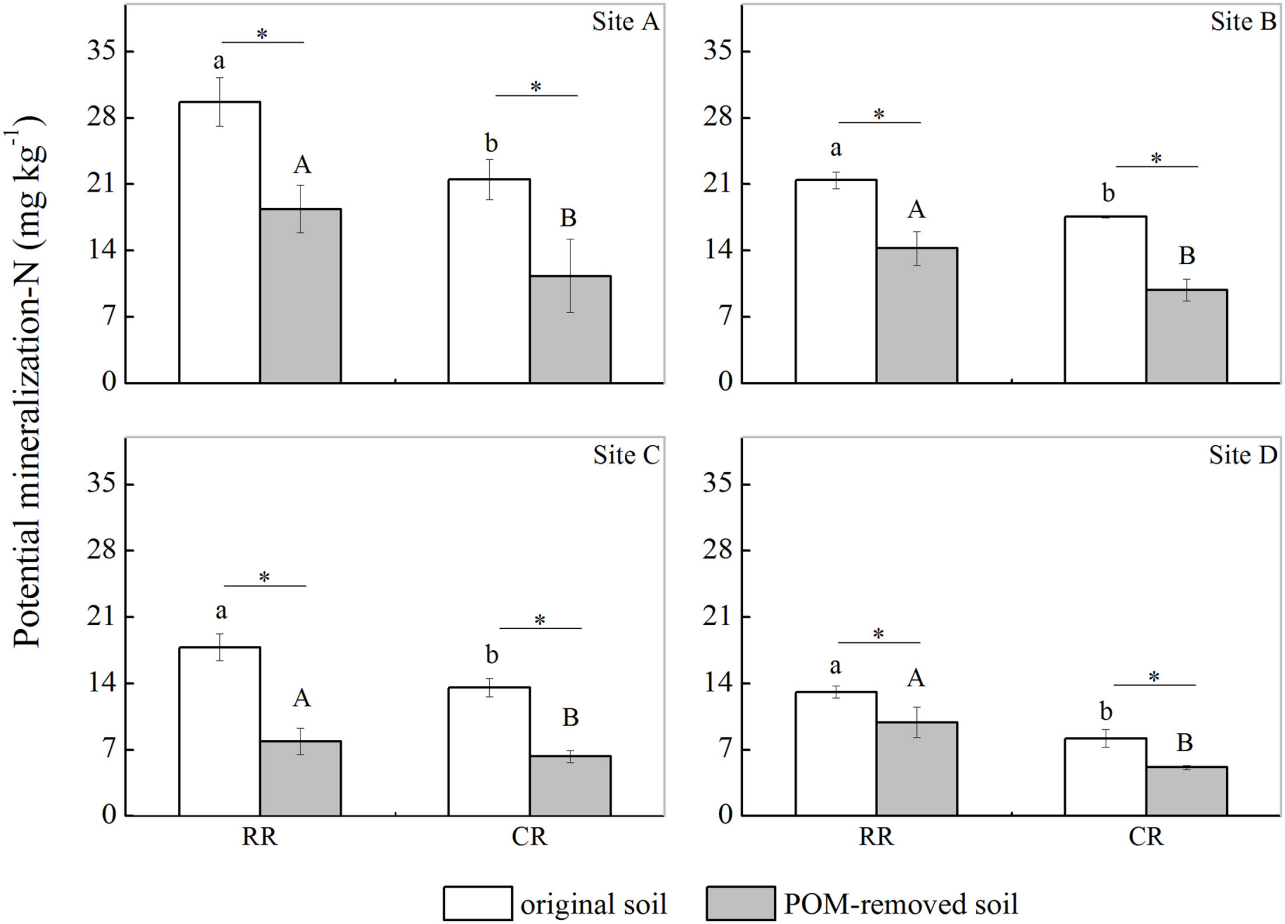
The effects of POM removal on the soil PMN contents under the different rotation systems. Note: RR corresponds to the rice-rapeseed rotation, and CR corresponds to the cotton-rapeseed rotation. Bars indicate the standard error of the mean (n = 2).

**Fig 4:**
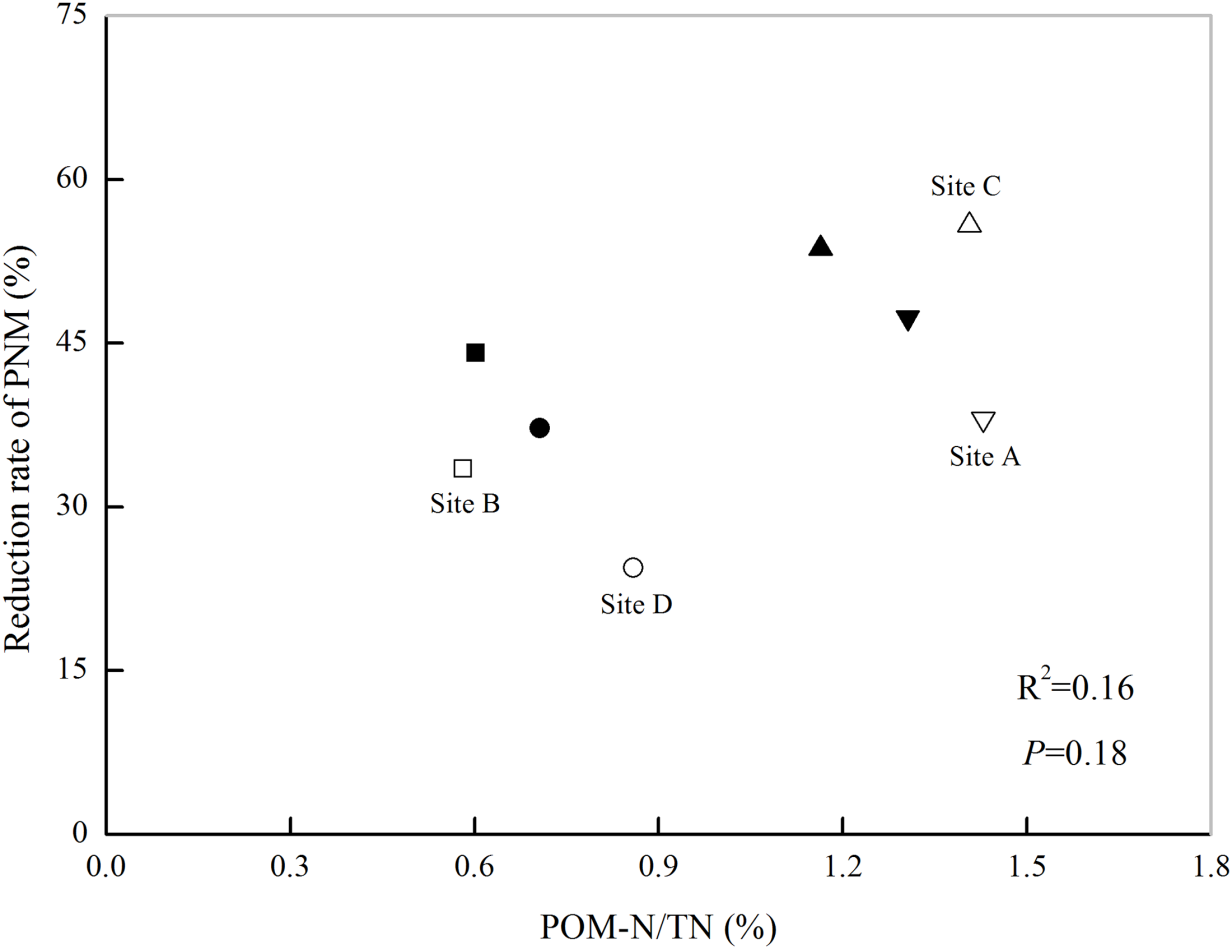
The relationships between the ratio of POM-N to TN and the reduction rate of PMN after POM-removal. Note: the hollow open represent the soils from the rice-rapeseed rotation and the solid symbols represent the soils from the cotton-rapeseed rotation. Reduction rate of PMN = (PMN_original soil_—PMN_POM-removal soil_) / PMN_original soil_ × 100.

Four paired soils representing different levels of soil PMN (Fig 1) were chosen to analyze the contributions of POM to soil N mineralization. It was found that POM removal significantly decreased the soil PMN content (*P* < 0.05, Fig 3, Table 3). Compared with the PMN content of the original soils, the PMN content of POM removal soils decreased by 11.29 mg kg^-1^, 7.18 mg kg^-1^, 9.96 mg kg^-1^, and 3.20 mg kg^-1^ at sites A, B, C, and D, respectively, under the RR rotation and by 10.19 mg kg^-1^, 7.76 mg kg^-1^, 7.29 mg kg^-1^, and 3.06 mg kg^-1^ at sites A, B, C, and D, respectively, under the CR rotation. Here, the rate of the PMN reduction after POM removal was defined as the contribution of the POM to the PMN. The contribution of the POM to the PMN varied from 24.4% to 55.8%, with an average of 37.9% under the RR rotation, whereas the average contribution rate was 45.6% under the CR rotation. The contribution of POM to PMN under the RR rotation was less than that under the CR rotation, except at site C, where the contributions of POM to PMN were 55.8% and 53.7% for the RR and CR rotations, respectively. No correlations were observed between the ratio of POM-N to TN and the reduction rate of PMN after POM removal (*P* = 0.18, Fig 4), indicating that the ratio of POM-N to TN could not fully explain the changes in the soil PMN after POM removal.

**Table 3:** Generalized linear model analysis of sources of variation of soil potentially mineralizable N (PMN) contents under two different rotations and particulate organic matter removal treatment.

Source of variation	Site A		Site B		Site C		Site D	
	*F*	*P* values	*F*	*P* values	*F*	*P* values	*F*	*P* values
Rotation (R)	21.585	0.002	47.519	< 0.001	19.312	0.002	70.451	< 0.001
POM removal (P)	42.879	< 0.001	155.01	< 0.001	167.658	< 0.001	29.512	0.001
R*P	0.114	0.745	0.236	0.64	4.008	0.08	0.016	0.904

### Effects of POM removal on microbial biomass size

The rotation had no effects on the soil MBC content. However, the level of MBN was greater under the RR rotation than under the CR rotation (Table 4). The removal of POM significantly decreased the soil MBC and MBN contents (*P* < 0.05). However, the MBN contents decreased more significantly than the MBC contents (*P* < 0.05). The corresponding soil MB C/N ratio was significantly increased after POM removal (*P* < 0.05). On average, the MBN content decreased by 16.3% and 19.5% and the MB C/N ratio increased by 13.9% and 20.0% under the RR and CR rotations, respectively. However, the reduction rate of soil MBN at site C under the RR rotation was higher than that under the CR rotation.

**Table 4:** Effect of POM removal on soil microbial biomass C, N and C/N ratio under the different rotation systems.

Site	Rotations	Microbial biomass C (mg kg^-1^)		Microbial biomass N (mg kg^-1^)		Microbial biomass C/N	
		Original soil	POM-removed soil	Original soil	POM-removed soil	Original soil	POM-removed soil
RR	A	635±38a	583±49a	**44.4±3.9**a	*35*.*8±3*.*4*ab	14.4±1.8a	16.3±0.7a
	B	612±22a	596±32a	**42.8±2.5**a	37.8±2.3a	14.4±1.3a	15.8±0.7ab
	C	525±24b	504±4b	*46*.*2±1*.*3*a	35.7±3.8ab	**11.4±0.8**b	14.3±1.6b
	D	515±16b	499±14b	*34*.*6±1*.*2*b	30.7±1.8b	**14.9±0.7**a	16.3±0.6a
CR	A	590±83a	540±83a	**35.9±4.4**ab	29.2±1.9ab	16.5±0.9a	18.5±1.7a
	B	594±22a	585±16a	**40.2±2.5**a	34.8±2.3a	**14.8±0.5**ab	16.8±0.8a
	C	515±19b	499±5b	**39.6±3.0**a	29.3±5.5ab	**13.1±1.4**b	17.5±3.3a
	D	505±18b	494±26b	**32.8±2.3**b	26.3±2.6b	**15.5±1.5**a	19.0±2.5a
***ANOVA***							
Rotation (R)		0.097		<0.001***		<0.001***	
POM removal (P)		0.034*		<0.001***		<0.001***	
Site (S)		<0.001***		<0.001***		0.001**	
R×P		0.833		0.901		0.221	
R×S		0.600		0.156		0.548	
P×S		0.550		0.127		0.417	
R×P×S		1.000		0.839		0.844	

Values followed by the same letters within a column are not significantly different (*P* < 0.05); values in bold are significantly different across the POM and POM-removal treatments; and values in italic are significantly different across the RR and CR rotations;

* denote significance at *P* < 0.05 level;

** denote significance at *P* < 0.01 level;

*** denote significance at *P* < 0.001 level.

**Fig 5:**
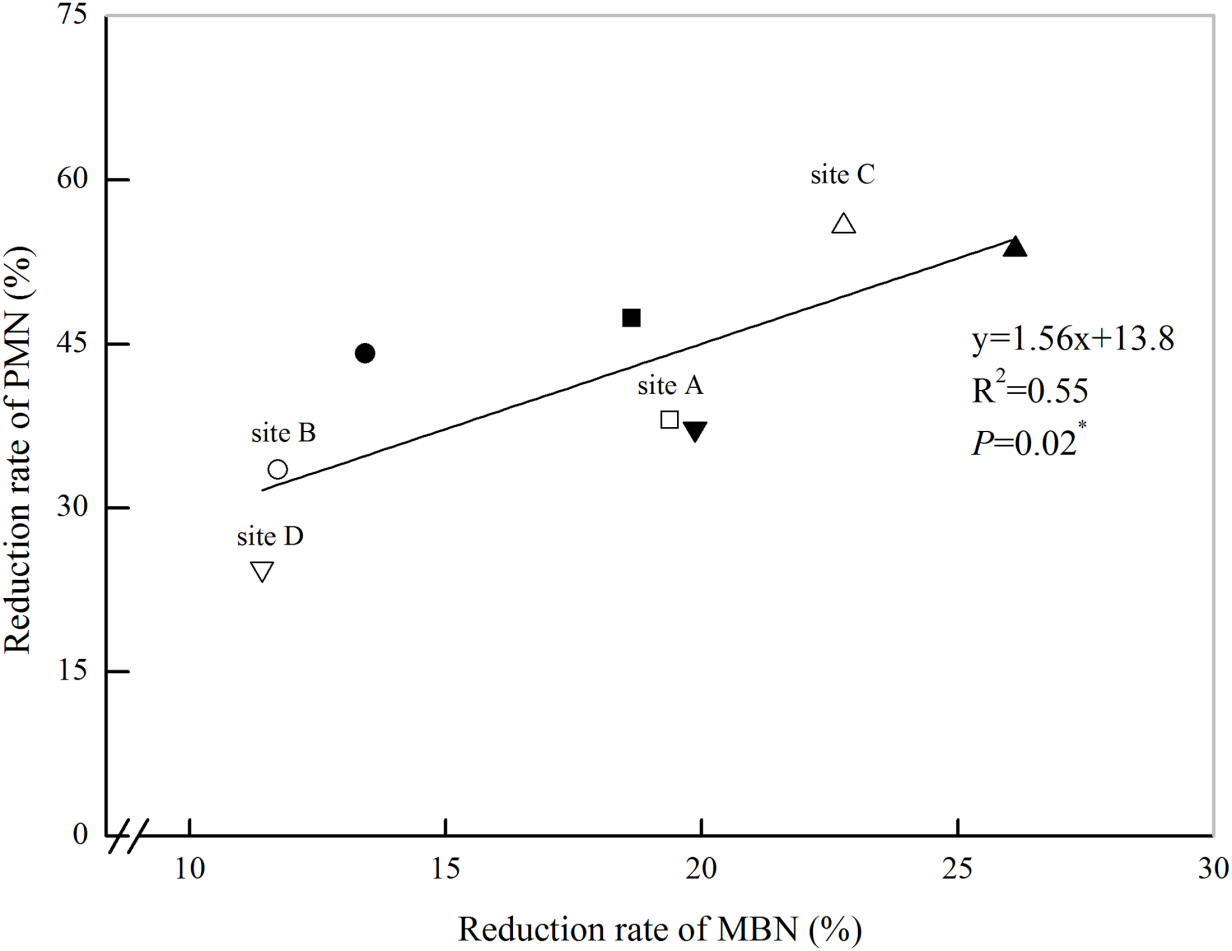
The relationships between the reduction rates of microbial biomass N (MBN) and PMN after POM-removal. Note: the open symbols represent the soils from the rice-rapeseed rotation and the solid symbols represent the soils from the cotton-rapeseed rotation. Reduction rate of MBN = (MBN_original soil_—MBN_POM-removal soil_)/MBN_original soil_ × 100; reduction rate of PMN = (PMN_original soil_—PMN_POM-removal soil_) / PMN_original soil_ × 100.

**Fig 6:**
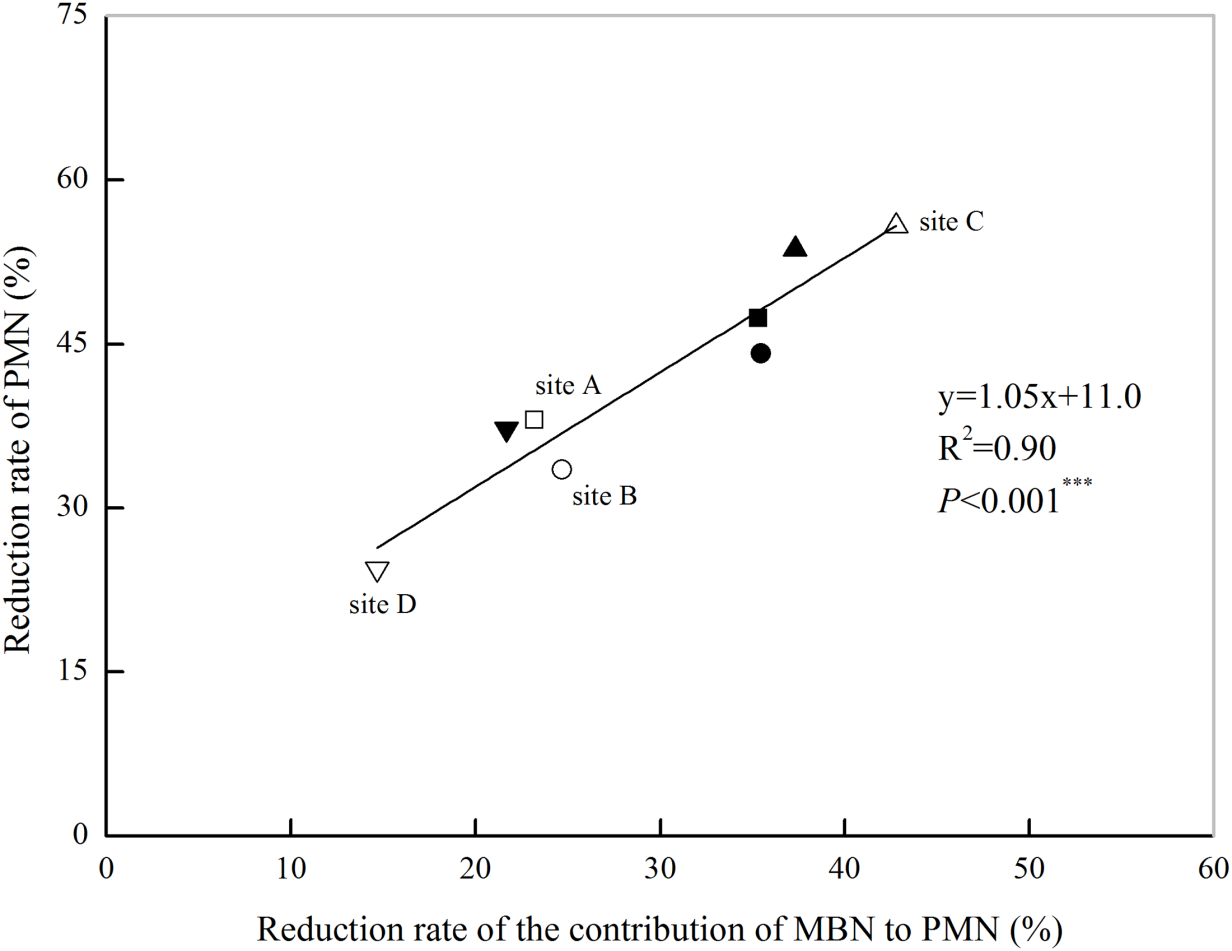
The relationships between the reduction rate of the contribution of MBN to PMN and the reduction rate of PMN content after POM-removal. Note: the open symbols represent the soils from the rice-rapeseed rotation and the solid symbols represent the soils from the cotton-rapeseed rotation. The contribution of soil MBN to PMN = MBN / PMN; decrease in the rate of contribution of MBN to PMN = (Contribution _original soil_—Contribution _POM-removal soil_) / Contribution _original soil_ × 100; Reduction rate of PMN = (PMN_original soil_—PMN_POM-removal soil_) / PMN_original soil_ × 100.

The soil MBN and PMN reduction rates after POM removal were significantly positively correlated (Fig 5). The decrease in soil PMN after POM removal reached a peak when the soil MBN reduction rate was highest, which indicated that the change in soil MBN was the main reason for the variation in soil PMN after POM removal because it accounted for 55% of the variation in the reduction of PMN. To quantify the relationships among the soil POM fractions, the soil MBN and the PMN, the contribution of soil MBN to PMN after POM was removed was examined (Table 5), this contribution was defined as the rate at which the contribution of MBN to PMN was reduced when POM was removed. For the original soils, the average contributions of the soil MBN to the PMN were 0.48 mg PMN mg^-1^ MBN and 0.40 mg PMN mg^-1^ MBN under the RR and CR rotations, respectively. When soil POM fraction was removed, the average values were 0.36 mg PMN mg^-1^ MBN and 0.28 mg PMN mg^-1^ MBN under the RR and CR rotations, respectively. The average reduction rates associated with soil POM removal were 25.3% and 31.2% under the RR and CR rotation, respectively. The high correlation between the reduced rate of the contribution of MBN to PMN and the reduction of PMN (*P*< 0.001, Fig 6) indicated that the changes in soil N mineralization were associated with the soil MBN induced by POM.

**Table 5:** Effect of POM removal on the contribution of soil microbial biomass N to soil potential mineralizable N under the different rotation systems.

Site	RR			CR		
	Original soil (mg mg^-1^)	POM-removed soil (mg mg^-1^)	Reduction rate (%)	Original soil (mg mg^-1^)	POM-removed soil (mg mg^-1^)	Reduction rate (%)
Site A	**0.64±0.04**a	0.52±0.04a	19.8	0.55±0.08a	0.41±0.23a	24.0
Site B	0.50±0.05b	*0*.*38±0*.*06*b	24.6	**0.44±0.03**b	0.28±0.01b	35.3
Site C	0.40±0.01c	0.22±0.05c	44.7	0.38±0.03b	0.22±0.01b	43.4
Site D	*0*.*38±0*.*02*c	*0*.*32±0*.*04*b	14.9	0.25±0.03c	0.20±0.03b	21.3
average	0.48	0.36	25.3	0.40	0.28	31.2
***ANOVA***						
Rotation (R)		<0.001***				
POM removal (P)		<0.00^1^ ***				
Site (S)		<0.001***				
R×P		0.967				
R×S		0.248				
P×S		0.212				
R×P×S		0.974				

The contribution of soil microbial N to potential mineralization N = microbial biomass N/soil potential mineralization N.

Values followed by the same letters within in a column are not significantly different (*P* < 0.05); values in bold are significantly different across the POM and POM-removal treatments; and values in italic are significantly different across the RR and CR rotation;

*** denote significance at *P* < 0.001 level.

### Infrared spectral features of the POM fraction

**Fig 7:**
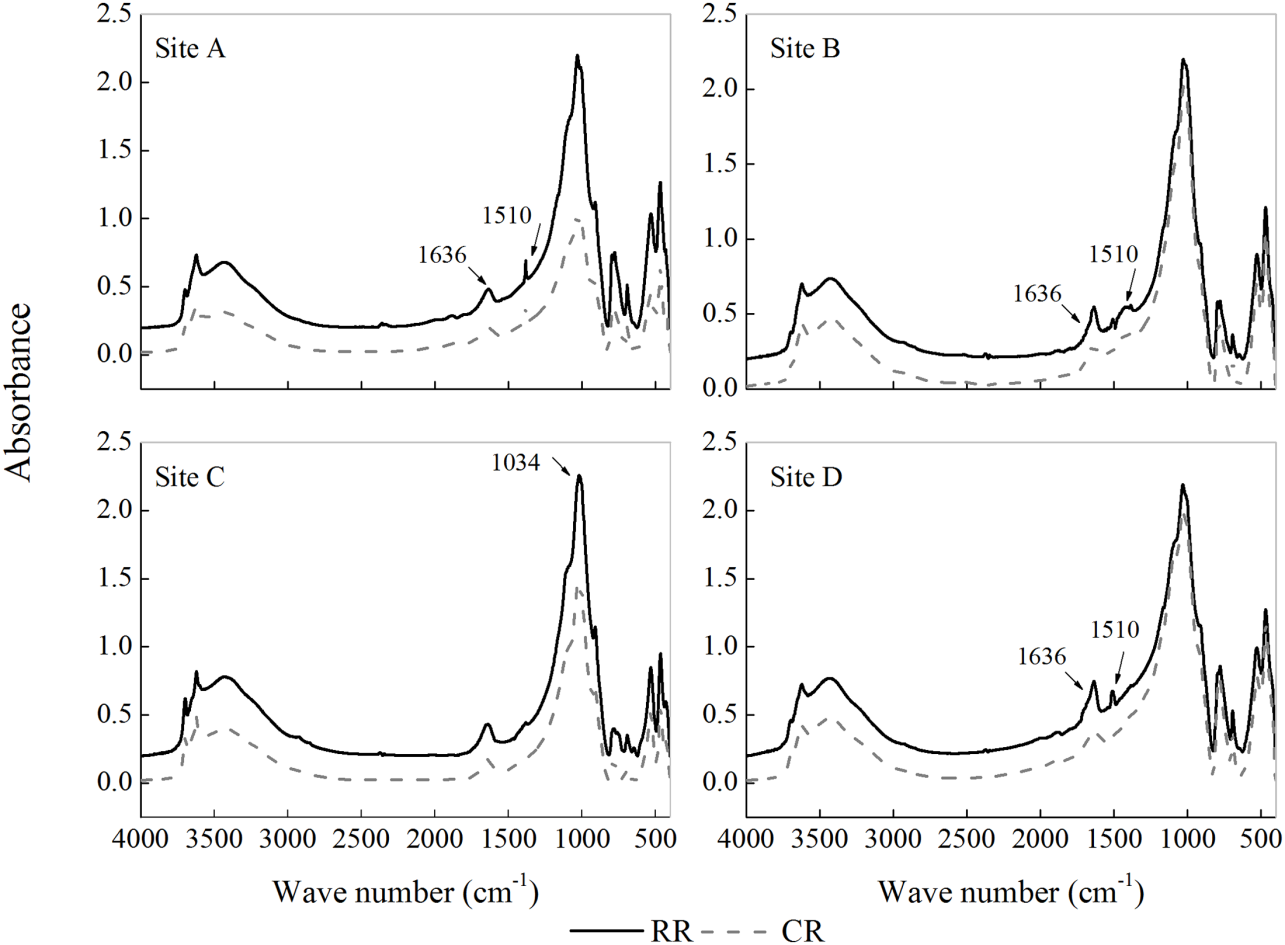
Infrared spectroscopy of soil POM under the different rotation systems. Note: RR corresponds to the rice-rapeseed rotation, and CR corresponds to the cotton-rapeseed rotation.

Neat samples of POM fractions derived from of the A, B, C and D sites exhibited specific spectral features (Fig 7). Differences in the soil POM spectral features were observed at 1636 cm^-1^, 1510 cm^-1^ and 1034 cm^-1^. The pronounced peak between 1600 cm^-1^ and 1650 cm^-1^ resulted from lignin and protein, and the peak at 1510 cm^-1^ was associated with the accumulation of aromatic recalcitrant materials. In addition, the peak at 1034 cm^-1^ resulted from carbohydrates and polysaccharides.

## Discussion

### The effects of the crop rotations on POM

It was found that the RR rotation resulted in a higher POM content than the CR rotation, which was consistent with previous studies [26]. In the present study, the samples were derived from paired plots within 1 km of each other with same soil parent material and similar in management history. Compared with CR rotation system, there were more crop residues in the soil in the RR rotation, which is the main factor influencing the accumulation of SOM [38]. Besides, soil environment *e*.*g*., repeatedly flooded in RR rotation, will reduce the decomposition of organic matter and increase the accumulation of organic matter [23]. Indeed, Gosling *et al*.[19] reported that the organic matter and the environmental factors (eg, temperature, moisture) is the primary factors controlling the POM content. Thus, we proposed that the difference in soil POM fractions was caused by the rotation systems. The alternating rotations were associated with different soil water and tillage management practices as well as differences in the crop residue quality and quantity, which finally resulted in significant differences in the SOM fractions [39, 40]. Shen *et al*. [30] mentioned that the quantity of crop residue inputs under the RR rotation was higher than under the CR rotation. Under the RR rotation, rice roots with a high C/N ratio were the main type of crop residues, whereas cotton leaves with a low C/N ratio were the main type of crop residue under the CR rotation [27]. Rice root tissue is less susceptible to decomposition by soil or litter microbes because of the greater quantities of lignin and tannin [41]. In addition, repeated transitions between flooding and drying could restrict the decomposition of organic matter and result in its accumulation in the soil [42]. The soil POM spectral features revealed that the RR rotation could promote the accumulation of low-bioavailability organic matter (mainly aromatic recalcitrant materials, Fig 7) in the POM fraction, with the exception of site C where more carbohydrates and polysaccharides other than aromatic recalcitrant materials showed increasing accumulation. The different trends observed at these four sites were presumably associated with the soil clay content, although we did not fully characterize the constituents of the POM. At site C, the soil clay contents under the RR and CR rotations were 18.4% and 17.2%, respectively, while the clay content was greater than 30% under the RR and CR rotations for all the other sites. In fact, soil clay content was an important factor that influenced the soil organic matter content, a higher clay content potentially contributed to more increased accumulation of soil organic matter [43, 44]. Therefore, we concluded that the RR rotation not only increased soil POM-C and POM-N contents but also changed the quality of the soil POM fraction via accumulation of decomposition-resistant plant residue and low-bioavailability organic matter.

### Effects of POM on soil N mineralization

POM is an important source of nutrients for microorganisms and has an important role in soil N mineralization [16]. The significant decrease in PMN after POM removal (Fig 3) suggested that POM has vital roles in soil N mineralization, indeed, it was previously reported that POM is a rapidly decomposable fraction and plays an important role in N turnover [45]. The soil POM-N content accounted for 22.7%-31.8% of the total soil N content. When the POM was removed, the soil PMN content was reduced by an average of 41.8%. Obviously, the ratio of POM-N to TN could not fully explain the changes in the soil PMN content when the soil POM was removed. Indeed, the soil POM fraction, which represented most of the decomposed plant residues in their early stages, was believed to be an easily accessible energy source for microorganisms [46]. Treatment with low-energy irradiation ultrasonic (50 J ml^-1^) at a low temperature (4°C) was not found to affect soil microbial activities [47]; furthermore the soil samples were pre-incubated at 25°C for 7 days to restore soil balance. Thus, the decrease in MBN was attributed to POM removal. In addition, Cookson *et al*. [12] confirmed that removing labile soil organic matter fractions influenced the soil microbial community and subsequently resulted in a decrease in the microbial biomass. The concurrent decrease in PMN and MBN with POM removal suggested that the decrease in PMN was associated with changes in MBN to some extent (Table 4); this was supported by the good correlation between the contributions of the MBN reduction rate to PMN and the rate of decrease PMN (Fig 6). In the present study, removal of POM by washing with water may have caused some dissolved organic matter (DOM) losses, which was thought to contribute 12.9%–20.0% of PMN [12, 48] and lead to an approximately 6% decrease in the MBN contents [12, 49]. However, the MBN content decreased by 16.3% and 19.5% under the RR and CR rotations, respectively, when POM was removed. These decreases are much larger than the average decrease (approximately 6%), implying that the decreases in PMN and MBN were mainly caused by POM removal.

The soil PMN contents decreased at four sites after POM was removed. At sites A, B, and D, the PMN reduction rates under the RR rotation were lower than those under the CR rotation. According to the spectral features of the soil POM, we speculated that several compounds with low bioavailability (*e*.*g*., lignin, aromatic recalcitrant material) increasingly accumulated in the soil POM fraction under the RR rotation, which reduced soil N mineralization [23]. By contrast, a peak representing carbohydrates and polysaccharides was observed in the soil under the RR rotation at site C, and accumulation of low-bioavailability compounds was not observed, leading to a greater decrease in soil PMN under the RR rotation than under the CR rotation. Overall, all the results support the hypothesis that the quality and/or quantity of soil labile organic matter is altered between and after the different cultivation systems, which could cause a decrease in the soil N supply under the RR rotation. The characteristic differences in the soil POM fraction could explain the variation in soil N mineralization after POM removal under the RR and CR rotations.

## Conclusions

The results of this research highlight the importance of the quantity and/or quality of POM for soil N mineralization under different rotation systems. Both C and N contents in POM were higher under the RR rotation than under the CR rotation. The PMN contents were closely related to the POM contents and appeared to be governed by intrinsic biochemical differences in the POM between the different rotation systems. Soil PMN and MBN contents at all the experimental sites were significantly decreased due to POM removal, and a greater reduction rate was found under the CR rotation compared with the RR rotation. Furthermore, infrared spectroscopy indicated that compounds with low bioavailability accumulated in the soil POM fraction under the RR rotation but not under the CR rotation. Therefore, we suppose that the information on quality and/or quantity of POM could be useful for predicting soil N mineralization to improve N fertilizer management in agricultural soils under different rotation systems. Soils under the RR rotation require more N fertilizer due to the low-bioavailability compounds accumulated in the soil POM fraction.

## Supporting Information

S1 Table Physical and chemical properties of tested soils from 16 sites. (XLSX)
